# Grandmothers matter: how grandmothers promote maternal perinatal mental health and child development

**DOI:** 10.3389/fpsyg.2024.1521335

**Published:** 2024-11-25

**Authors:** Madelon M. E. Riem, Merel van der Straaten

**Affiliations:** ^1^Behavioural Science Institute, Radboud University, Nijmegen, Netherlands; ^2^Department of Psychology, University of Cambridge, Cambridge, United Kingdom; ^3^Department of Neonatology, Wilhelmina Children’s Hospital, University Medical Center Utrecht, Utrecht, Netherlands

**Keywords:** maternal mental health, perinatal depression, grandparental support, grandmother, child development, caregiving

## Abstract

Low social support has been identified as a risk factor for maternal perinatal mental health problems. However, previous studies have predominantly focused on general social support or support from the partner, often overlooking the roles of grandparents. This mini review discusses recent developments in perinatal health research showing that supportive grandparents may constitute a protective factor against the development of maternal perinatal mental health problems. In addition, we will discuss how grandparental support may promote fetal and child health. The mini review concludes with a call for more research on the role of grandparents in perinatal health. Recognizing grandmothers as collaborators in maternal and child health may afford more effective perinatal health programs and clinical practice, thereby reducing the risk of adaptational and developmental problems.

## 1 Introduction

The traditional African proverb “It takes a village to raise a child” may express an underlying truth. Mothers, or fathers, do not rear children on their own, but across many cultures around the world, they share childcare with co-parents, grandparents, older siblings, aunts, and communities. These substantial contributions from “*allomothers*” to childcare can be explained from an evolutionary approach in which humans are characterized as *cooperative breeders*, meaning that parents do not raise their children on their own but are assisted by members of their social group (Crittenden and Marlowe, 2008; Hawkes, 2004; Hrdy, 2009; Kramer, 2010; Meehan et al., 2014). According to anthropology research, allomothers who assist the mother–child dyad are especially important during the intensive first years after birth when new mothers are most in need of support (Hrdy, 2009). Stern’s theory on the ‘motherhood constellation’ also suggests that mothers-to-be experience an attentional shift during pregnancy, with a new focus on the all-consuming role of mother and the support systems that are needed to meet the needs of motherhood responsibilities (Stern, 1995). In particular grandparents, most importantly the maternal grandmother (Coall et al., 2018; Perry and Daly, 2017; Riem et al., 2023), are a source of emotional and practical support during pregnancy and the postpartum period (Aubel, 2021; Coall et al., 2018). After child birth, grandmothers may provide advice on infant care (Aubel, 2021), breastfeeding (Negin et al., 2016) or practical assistance with childcare when mothers return to work (Kanji, 2018), thereby promoting adjustment to motherhood.

Although beneficial influences of partner support are well-documented (Yim et al., 2015), the role of supportive grandparents in preventing perinatal mental health problems has received less attention. Despite the important role of grandmothers in providing support to new mothers, perinatal health research has neglected the role of grandparents in maternal mental health. This mini review aims to address this ‘grandparent gap’ in perinatal health research. The review discusses how grandparents promote maternal perinatal mental health by offering emotional and practical support during pregnancy and the postpartum period. Additionally, we discuss how grandparental support directed toward the pregnant woman may beneficially impact fetal development. As conflicts with grandparents may compromise maternal mental health, the review also addresses a controverse by discussing both the challenges and the benefits of grandparental support. Importantly, resolving intergenerational conflict and promoting high-quality support from grandparents by involving them in treatment may add to the effectiveness of existing perinatal mental health interventions. This mini review therefore concludes with a call for more research on the role of grandparents in perinatal health.

## 2 The grandmother as a valuable allomother

Human parenting is intensive. Human infants are helpless after birth, fully dependent on their caregivers, and they require large parental investments during a long period of childhood. Evolutionary theorists suggest that such intensive investment is only possible because childcare is shared among multiple caregivers, the allomothers (Crittenden and Marlowe, 2008; Hawkes, 2004; Hrdy, 2009; Kramer, 2010; Meehan et al., 2014). Grandmothers, in particular, have been described as highly valuable allomothers due to their reproductive experience and expertise (Knorr and Fox, 2023). They assist mothers in various ways, such as during childbirth, the weaning period (Rosenberg and Trevathan, 2002), or with breastfeeding (Scelza and Hinde, 2019). Research on pre-industrialized societies indeed suggests that grandmaternal support ‘lightens the reproductive load’ and allows parents to reproduce earlier, more frequently and more successfully. More specifically, using complete multi-generational demographic records of pre-modern populations of both Finns and Canadians, Lahdenperä et al. (2004) showed that women with a prolonged post-reproductive lifespan have more grandchildren and hence greater fitness. Other studies also indicate an association between presence of the maternal grandmother and improved child survival (Fox et al., 2010; Sear and Mace, 2008).

Grandparental support should not be considered a “relic of the past” (Coall and Hertwig, 2011). In modern societies, where life expectancy and rates of single motherhood and female employment are increasing, grandparents continue to play a crucial role in the caregiving puzzle. Research even shows that more grandparents are involved in care for grandchildren than at any other time in recent history (Buchanan and Rotkirch, 2018). The likelihood that a child lives with grandparents has nearly quadrupled from 7% in 2011 to 26% in 2021 in the Unites States.^1^ Although both grandparents contribute to childcare, grandmothers are more likely to provide care, in particular the maternal grandmother (Danielsbacka et al., 2011; Fuller-Thomson and Minkler, 2001; Minkler and Fuller-Thomson, 2005; Wheelock and Jones, 2002). A study of 10 European countries showed that 58% of grandmothers looked after a grandchild (Hank and Buber, 2009). Grandmothers may not only offer help with childcare, but may also provide new mothers with emotional, financial, and informational support during the perinatal period (Aubel, 2021; Burgess, 2015; Zartler et al., 2021), thereby enhancing the mother’s mental health and energy balance, promoting the bond between mother and infant, and improving the child’s nutritional outcomes (Scelza and Hinde, 2019). These important grandmaternal support roles substantiate the claim in anthropology literature that humans have evolved in an adaptive sociocultural perinatal complex in which grandmothers have a unique role and provide crucial contributions to the well-being of their daughters and grandchildren (Scelza and Hinde, 2019).

## 3 Grandparents as a source of postpartum support for mothers

Pregnancy and the first months after birth is a period of vulnerability for the onset of maternal affective disorders, particularly depression affecting 10–15% of all mothers in Western societies (Gavin et al., 2005; Pop et al., 2019). Lack of support from a partner, family and friends has been shown to increase risk for maternal affective disorders, in particular postpartum depression (Cho et al., 2022; Hetherington et al., 2018; Leahy-Warren et al., 2012). According to the adaptationist hypothesis for postpartum depression (PPD), PPD may be even a strategic adaptation to a lack of a support system for mothers (Hagen, 1999), meaning that receiving insufficient support necessary for raising a child may trigger a depressive state in mothers and consequently low maternal investment in the child. This low investment, in turn, elicits greater levels of support and investment from partners, grandmothers, and/or other relatives. Indeed, it has been shown that grandparents step in and take on child caregiving responsibilities in response to maternal mental illness or other family problems (Fuller-Thomson and Minkler, 2001; Hayslip et al., 2014; Jackson et al., 2023; Riem et al., 2025).

Although the role of the support system in postpartum mental health is well-documented, less attention has been devoted to support provided by grandparents during the first year postpartum. The results of studies examining the association between grandparental presence or co-residence and maternal mental health in the first year postpartum are somewhat mixed. For instance, Arnold et al. (2011) found that sharing childcare with multiple family members, including grandparents, promotes maternal mental health. Mothers felt more supported when grandparents are involved in childcare. However, the authors reported no differences in parenting stress between mothers who received practical childcare support from grandparents and those who did not. Other studies examining the association between maternal mental health and grandparental co-residence indicate lower levels of depressive symptoms at 6 months postpartum in mothers with co-residing grandparents, although dependent on the family cohesion (Kalil et al., 1998), while findings from Way and Leadbeater (1999) observed no significant association. Interestingly, living with parents-in-law may even increase risk of PPD for mothers, as indicated by Wang et al. (2017) in a Chinese sample. Hence, childcare assistance from grandparents seems to promote maternal mental health, although the genetic relationship with grandparent and the quality of the relationship matters.

Several studies have examined how quantity and/or quality of grandparental care is related to maternal postpartum mental health. In a sample of adolescent mothers, Borcherding et al. (2005) found that mothers who received high quality grandparental support experienced fewer depressive symptoms. Another study showed that support from the maternal grandmother was significantly related to better maternal mental health among Israeli mothers with preterm twins, but not among mothers with full-term twins (Findler et al., 2007). In a sample that partially overlapped with the previous study and was overrepresented with mothers of twins or (singleton) preterm infants, the authors (Noy et al., 2015) found that both grandmother’s instrumental and emotional support were significantly related to better maternal postpartum mental health. Leadbeater and Linares (1992) also showed that higher quality of the grandmother-daughter relationship was negatively related to mothers’ depressive symptoms in a sample of adolescent mothers. Similarly, Lee et al. (2020) showed a negative association between emotional support from grandparents and parenting stress in African-American mothers. In the study conducted by Leung and Lam (2012), effects of grandparental involvement on maternal mental health were examined with a randomized controlled trial. An antenatal group intervention targeting intergenerational conflicts with grandparents resulted in a reduction of depressive symptoms over time, indicating that resolving conflicts about childcare with grandparents reduces stress and depressive symptoms in mothers. Hence, emotional and practical grandparental support may have beneficial effects for mothers’ mental health, although quality of support seems to matter.

In a recent meta-analysis, including 11 empirical studies (*N* = 3,381 participants), we examined the association between grandparental support and maternal mental health during the first year postpartum (Riem et al., 2023). We found that a higher level of grandparental support, in particular from the maternal grandmother, is related to better maternal postpartum mental health. In another recent study, we examined how grandparental support is related to depressive symptoms in a clinical sample of mothers diagnosed with major depression (Riem et al., 2024). The results suggested that higher perceived grandparental support was related to less severe maternal depressive symptoms at 12 months postpartum. This indicates that supportive grandparents may prevent the development of more severe PPD in mothers experiencing mental health problems. Interestingly, our study also showed that practical grandparental support with childcare was not related to severity of PPD, possibly because the quality of the intergenerational relationship matters for mothers. Intergenerational conflicts may add to family stress, thereby negatively impacting on maternal mental health (Lau and Keung Wong, 2008).

## 4 Prenatal grandparent effects on the mother and the developing child

The majority of grandparenting studies have focused on grandparents as a source of support during the postpartum period. However, less is known about the role of grandparents during pregnancy. Pregnancy represents a period of plasticity and vulnerability during which a high-quality social support network can have beneficial influences on offspring health. Environmental influences on fetal development during pregnancy have been described in the Developmental Origins of Health and Disease model (DOHaD) (Langley-Evans and McMullen, 2010), which posits that environmental exposers early in life, in particular during the prenatal period, may lead to fetal adaptations that have long-term health implications. Supporting the DOHaD model, a large number of studies suggest that maternal psychological distress during and after pregnancy predicts poor neurodevelopmental outcomes in children (Girchenko et al., 2018; Manzari et al., 2019; Pearson et al., 2016; Van den Bergh et al., 2020). Other studies indicate that maternal psychological distress is related to maternal biomarkers, including higher levels of cortisol, C-reactive protein, homocysteine, interleukin-6, and lower levels of vitamin D, zinc, and anti-inflammatory markers (Serati et al., 2016), possibly underlying the association between maternal prenatal distress and poor infant outcomes. Although protective factors have received less attention in the DOHaD model, recent research indicates that general perceived social support (not specifically from grandparents) during pregnancy is associated with better child cognitive ability (Lähdepuro et al., 2024). Hence, support networks may buffer both mother and fetus from adaptational and developmental problems.

In contrast to the grandparental roles in providing postpartum support, few studies examined the influences of grandparents during pregnancy. Under the DOHaD framework, grandparents may invest in the prenatal period, offering support to the new mother-to-be, in order to increase their inclusive fitness. Grandparents may offer emotional support for the mother-to-be or offer practical support and diminish mother’s physical labor by relieving her of household chores or care for other children. In a recent study, Knorr and Fox (2023) were one of the first to examine how grandparental support relates to maternal prenatal mental health. They found that greater communication with and social support from the maternal grandmother was associated with less depressive symptoms in a sample of pregnant (majority in third trimesters) Latina women in the US. Interestingly, support from the paternal grandmother was not related to maternal mental health. This is in line with our meta-analysis showing that in particular support from the maternal grandmother is related to better maternal postpartum mental health (Riem et al., 2023). Perhaps, maternal grandmothers invest more because they are more certain about their genetic relationship with the grandchild or because they feel more emotionally connected to mother. Research suggests that the quality of attachment relationships is relatively stable across generations, e.g., from grandmothers to mothers to grandchildren (Hautamaki et al., 2010). Hence, grandmothers may continue their role as secure base for their adult daughters, particularly in times of need.

In another study, Fox et al. (2023) examined the endocrine mechanism by which prenatal grandmother effects may be enacted. They found that mothers who received more support from the maternal grandparental had lower cortisol levels during pregnancy. More specifically, the quality of the relationship between the grandmother and the mother-to-be, social support received from grandmother, and frequency of seeing grandmother were negatively associated mother’s urinary cortisol levels. Again, when comparing paternal versus maternal grandmothers, effects of the maternal grandmother were more pronounced. Other studies have shown that higher cortisol levels are associated with greater risk of preterm birth (Giurgescu, 2009) and correlate with fetal cortisol (Gitau et al., 2004). Hence, under the DOHad framework, lower cortisol may be one mechanism underlying grandmother intergenerational effects on child developmental outcomes (Sadruddin et al., 2019). However, it should be noted that no causal conclusions can be drawn with regard to the direction of the association between grandparental support and maternal cortisol as this study was cross-sectional. It is plausible to assert that low grandmaternal support increases maternal distress and cortisol, however, associations could be bi-directional, with worse mental health and higher cortisol causing pregnant women to perceive their grandmother relationship in a more negative way. Moreover, maternal mental health problems may hinder mothers in mobilizing caregiving support (Feinberg, 2003; Negron et al., 2013). Hence, although these pioneering studies on grandparent effects during pregnancy point to associations between grandparental support and maternal prenatal health and possibly fetal development, longitudinal studies or interventions manipulating quality of grandparental involvement are needed to test the direction of the association between grandparental support and maternal and child outcomes.

## 5 Grandmothers buffering effects of postpartum depression on child development

During the postpartum period, grandmothers may continue their safeguarding role and prevent or diminish the adverse effects of maternal mental health problems on the developing child. PPD can disrupt maternal sensitivity (Bernard et al., 2018), defined as the ability to respond to and interpret child signals in a correct way and respond adequately and promptly (Ainsworth, 1978). In turn, this increases risk for child attachment insecurity and poor socio-emotional development (De Wolff and van Ijzendoorn, 1997; Deans, 2020). Grandparents may, however, temper the threat of impaired parenting of depressed mothers. For instance, our recent study showed that higher perceived grandparental support is related to less parenting stress 1 year after childbirth in mothers with PPD (Riem et al., 2024), indicating that supportive grandparents might protect against parenting difficulties in depressed mothers. Silverstein and Ruiz (2006) also showed that grandparents function as compensatory family resources who safeguard their grandchildren from adverse risks associated with maternal depression. More specifically, grandchildren who were least integrated with their grandparents resembled their mothers in the severity of depressive symptoms as young adults.

Grandparental promotive effects on child development may be indirect, mediated by a higher-quality of maternal care and lowered parenting stress, or direct, resulting from interactions in which grandparents serve as secondary caregivers. Grandparents may take on important roles such as attachment figure (Liang et al., 2021) or offering emotional support resources to the child, thereby promoting child development. This may be particularly important for children who grow up under adverse circumstances, such as maternal mental illness. Indeed, studies examining the effects of cumulative adverse early life events beyond the postpartum period point to protective effects of grandparents on child development (Flouri et al., 2010; Riem et al., 2025), in particular when children receive support from the maternal grandmother (Helle et al., 2024). Together, these studies underscore the important role of grandparents in buffering negative effects of perinatal depression on child development and indicate that the current exclusive focus on parental caregiving roles in perinatal health research and parenting studies is insufficient.

## 6 Discussion

This mini review summarized findings showing that supportive grandparents may constitute a protective factor against the development of maternal perinatal mental health problems. By applying the DOHaD framework, the review discussed how grandparental support beneficially affects fetal and child development. Although there is a lack of attention to the role of grandparents in perinatal health research, studies conducted so far suggest that grandparents can *directly* promote better child development in the case of maternal mental illness, by offering love and support to the child, or *indirectly*, through reduced maternal prenatal and postnatal caregiving stress. In particular, support from the maternal grandmother is a protective factor against the development of perinatal depression, and associated parenting difficulties and risks for the developing child. However, research conducted so far is mostly cross-sectional. Longitudinal designs and/or interventions manipulating quality of grandparental care are urgently needed to test causal grandparent effects on maternal mental health. Moreover, more research is needed on the role of type of grandparent (paternal versus maternal grandparents and grandfathers versus grandmothers).

Despite the protective effects of grandmothers, the quality of the intergenerational relationship may matter, as conflicts may diminish benefits of grandmaternal support. Future studies should therefore examine whether resolving conflicts and stimulating high-quality support from grandmothers, for example by involving grandmothers in treatment, adds to the effectiveness of existing perinatal mental health interventions. Previous studies have shown that involving the mother’s partner in treatment for perinatal mood problems brings benefits for mother’s recovery (for a review see Pilkington et al., 2015). However, there is a lack of intergenerational perinatal mental health interventions and it is unknown whether involving grandparents in treatment offers benefits. Given recent reports on the rise of maternal mental health problems in Western societies (Pop et al., 2019), an intervention enhancing quality of grandparental support could be a new and urgently needed avenue for enhancing maternal perinatal mental health and child development.

## Footnotes

^1^ https://www.gu.org/
